# Exosome secreted from adipose-derived stem cells attenuates diabetic nephropathy by promoting autophagy flux and inhibiting apoptosis in podocyte

**DOI:** 10.1186/s13287-019-1177-1

**Published:** 2019-03-15

**Authors:** Juan Jin, Yifen Shi, Jianguang Gong, Li Zhao, Yiwen Li, Qiang He, He Huang

**Affiliations:** 1 Bone Marrow Transplantation Center, The First Affiliated Hospital, Zhejiang University School of Medicine, Hangzhou, Zhejiang 310003 People’s Republic of China; 2 Department of Nephrology, Zhejiang Provincial People’s Hospital, Hangzhou, Zhejiang 310014 People’s Republic of China; 3 People’s Hospital of Hangzhou Medical College, Hangzhou, Zhejiang 310014 People’s Republic of China; 4 Department of hematology, The First Affiliated Hospital, Wenzhou Medical University, Fuxue Road 2th, Wenzhou, 325000 People’s Republic of China

**Keywords:** Adipose-derived stem cells, Exosome, Podocyte damage, Cell autophagy, miR-486, Smad1/mTOR signaling

## Abstract

**Background:**

It is confirmed that adipose-derived stem cells (ADSCs) transplantation effectively relieves kidney fibrosis and type 2 diabetes disease in mice. Currently, exosome from urine-derived stem cells (USCs) can protect type 1 diabetes-mediated kidney injury and attenuate podocyte damage in diabetic nephropathy (DN). Exosome derived from USCs has evolved into the strategy for DN treatment, but the role of ADSCs-derived exosome (ADSCs-Exo) in DN remains unclear. The present study is aimed to investigate the therapeutic action and molecular mechanism of ADSCs-derived exosome on DN.

**Methods:**

ADSCs and exosome were authenticated by immunofluorescence and flow cytometry. Morphology and the number of exosome were evaluated by electron microscope and Nanosight Tracking Analysis (NTA), respectively. Cell apoptosis was assessed using flow cytometry. Podocyte autophagy and signaling transduction were measured by immunofluorescence and immunoblotting. Dual Luciferase Reporter assay was employed to detect the regulatory relationship between miR-486 and Smad1.

**Results:**

ADSCs-Exo attenuated spontaneous diabetes by reducing levels of urine protein, serum creatinine (Scr), blood urea nitrogen (BUN), and podocyte apoptosis in mice. In in vitro experiment, ADSCs-Exo also reversed high glucose-induced decrease of cell viability and the increase of cell apoptosis in MPC5 cells. In terms of mechanism, ADSCs-Exo could enhance autophagy flux and reduce podocyte injury by inhibiting the activation of mTOR signaling in MPC5 and spontaneous diabetic mice. Eventually, we found that miR-486 was the key factors in ADSCs and in the process of ADSCs-Exo-mediated improvement of DN symptom in vivo and in vitro. miR-486 reduced Smad1 expression by target regulating Smad1 whose reduction could inhibit mTOR activation, leading to the increase of autophagy and the reduction of podocyte apoptosis.

**Conclusions:**

In conclusion, we illustrated that ADSCs-Exo vividly ameliorated DN symptom by enhancing the expression of miR-486 which led to the inhibition of Smad1/mTOR signaling pathway in podocyte. Possibly, ADSCs-Exo was used as a main therapeutic strategy for DN in future.

**Electronic supplementary material:**

The online version of this article (10.1186/s13287-019-1177-1) contains supplementary material, which is available to authorized users.

## Background

Diabetic nephropathy (DN) is a complex and common chronic kidney disease worldwide [1]. The clinical features of DN are characterized by elevation of persistent albuminuria, thickness of glomerular basement membrane (GBM), and aggregation of the extracellular matrix, which results in autophagy flux inhibition, podocyte damage promotion, and progressive renal dysfunction [2]. However, the pathogenesis of podocyte injury-mediated DN remains vague and therapeutic strategies for DN are lacking.

Podocyte damage is a key regulator and can be used as a clinical predictor in the evolution of DN [3, 4]. Homeostasis maintenance under pathophysiological stress is extremely important in determining the fate of podocytes [5]. Podocyte autophagy is mainly responsible for protein and organelle degradation following by regulating cell homeostasis, whose dysfunction is regarded as an essential induction factor of podocyte injury [6, 7]. Numerous signaling molecules are involved in the process of podocyte autophagy including mTOR (mechanistic target of rapamycin) and AMP-activated protein kinase (AMPK) [8, 9]. In fact, mTOR signaling activation is closely linked to the accelerated podocyte injury in DN patients which potentially suggests an involvement of mTOR signaling-mediated autophagy dysfunction in DN disease [10, 11]. Collectively, autophagy flux might be a therapeutic target in podocyte damage-derived DN disease.

Previous studies have demonstrated that mesenchymal stem cells (MSCs) are a therapeutic candidate for halting the progression of DN [12]. There have been identified that adipose-derived stem cells (ADSCs) transplantation can alleviate acute kidney injury by enhancing cell repair and regeneration abilities [13]. ADSCs transplantation also attenuates podocyte damage in DN by activating klotho and inhibiting Wnt/β-catenin pathway [14]. Though many researchers have confirmed that ADSCs possesses the repair effect on cell/tissue injury, these protective effect is depended on the paracrine of ADSCs due to the low rate of effector cells differentiated from ADSCs after transplantation. Paracrine cytokines and exosome derived from ADSCs act as the key repair factors for wound healing [15, 16]. Especially, ADSCs-derived exosome (ADSCs-Exo) are internalized into fibroblasts to improve cutaneous wound healing by promoting cell migration, proliferation, and collagen synthesis [17]. Besides, exosome secreted by urine-derived stem cells have the potential to promote renal repair by repressing podocyte apoptosis in diabetic rats [18]. These studies suggest that exosome secreted from stem cells is the key paracrine regulator for cell/tissue repair. However, whether ADSCs-Exo possesses the protective effect on podocyte injury in DN and acts as a bridge between autophagy and apoptosis in podocyte damage are still not clearly understood.

Herein, ADSCs and ADSCs-Exo were isolated successfully from C57BL/KsJ db/m mice and ADSCs-Exo administration reduced cell apoptosis through regulating mTOR signaling-mediated autophagy flux in DN mice and high glucose-induced MPC5 cells. Mechanismly, miR-486 of ADSCs-Exo acted the key improvement role on renal injury through weakening mTOR activation-mediated autophagy via directly targeting smad1. Possibly, ADSCs-Exo might be served as a main treatment strategy for DN in future.

## Materials and methods

### Materials

Mannitol (SM8120) was obtained from Solarbio. RPMI-1640 (SH30809.01B), DMEM/F12 medium (SH30023.01B), and BSA (SH30574.03) were purchased from Hyclone. Collagenases I (17100-017), II (17101-015), IV (17104-019), and 0.25% Trypsin (15050065) were purchased from Gibco. PI (25535-16-4), RNase A (9001-99-4), dexamethasone (D4902), β-glycerophosphoric acid (G9422), ascorbic acid (A7562), insulin (I5500), indomethacin (I7378), and 3 isobutyl 1 methylxanthine (IBMX; I7018) were from Sigma. 1% Triton x-100 was obtained from SBJBio Life Sciences (SBJ-1141).

### Animal treatment

Eight-week-old C57BL/KsJ db/m (control mice, *n* = 10) and C57BL/KsJ db/db (spontaneous diabetes mice, *n* = 20) male mice were obtained from Cavens Lab Animal (SCX, 2016-0010, Changzhou, China). All animal care procedures were approved by Laboratory Animals of Zhejiang Provincial People’s Hospital. The mice were housed three to five per cage in a room under controlled light (12 h/day) and temperature (22 ± 2 °C) conditions with free access to food and water. At 12 weeks old, periodic acid-Schiff (PAS) staining was performed to for histopathology investigation to verify disease phenotype of DN according to previous study. At 13 weeks old, mice were treated with tail intravenous injection of PBS or ADSCs-Exo for additional 12 weeks.

### Cell isolation, culture, and treatment

Eight-week-old C57BL/KsJ db/m male mice was anesthetized using pentobarbital (200 mg/kg i.p.), and subcutaneous adipose tissue in groin was harvested. ADSCs were isolated by utilizing collagenase digestion method. Briefly, adipose tissue was minced and washed with PBS buffer twice followed by centrifugation at 1200 rpm for 10 min. The supernatant was removed, and the mixed collagenases were added into the precipitate. After digestion for 40 min at 37 °C, complete medium was added to stop the reaction and the digested mixture was filtered through 40-mm cell strainer and centrifuged at 1500 rpm for 8 min. The precipitate was then resuspended in RPMI-1640 complete medium and cultured at 37 °C in a humidified incubator in 5% CO_2_. In miRNA-486 inhibitor assay, ADSC cells were transfected with miRNA-486 inhibitor and negative control in ADSCs for 48 h. Then, ADSCs-Exo were isolated for the next experiments. The sequences of miRNA-486 inhibitor and negative control were as follows: AUCCUGUACUGAGCUGCCCCG and CAG UAC UUU UGU GUA GUA CAA.

Mouse podocyte MPC5 cells were purchased from Institute of Basic Medical Sciences, Chinese Academy of Medical Sciences (3111C0001CCC000230). MPC5 cells were cultured in DMEM medium containing with 10% fetal bovine serum and incubated at 37 °C in a humidified atmosphere containing 5% CO2. For CCK8 assay, MPC5 cells were treated with 5.5 mM d-glucose (NG), 5.5 mM d-glucose+ 24.5 mM mannitol (MA), 30 mM d-glucose (HG), and combination of HG and concentration gradient ADSCs-Exo for 24 h, 48 h, 72 h, and 96 h. For apoptosis and autophagy experiments, MPC5 cells were treated with NG, MA, HG, and combination of HG and ADSCs-Exo for 48 h.

### Osteogenic and adipogenic differentiation and authentication of ADSCs

ADSCs in passage 3 were subjected to stem cell induction and appraisal system for examining differentiation ability of ADSCs. For the ability of osteogenic differentiation, 1 × 10^5^ cells were seeded on coverslip in six-well plate. After incubation for 24 h, cells were induced with osteogenic medium containing 10% FBS, 10 nM dexamethasone, 10 mM β-glycerophosphate, and 50 μg/mL ascorbic acid in high-glucose DMEM medium. Normal high-glucose DMEM was served as the control group. At day 14, the induced cells were harvested for adipogenic assessments by utilizing alkaline phosphatase (ALP) detection kit (A059-2, Jiangcheng biotechnology, Nanjing, China) and Alizarin Red Staining. The ability of ALP was according to the manufacturer’s instruction. ALP activity was measured at the wavelength of 520 nm, and activity relative to control was calculated after normalization to the total protein content. Alizarin Red Staining was another employed method for detecting mineralization after osteogenic differentiation. In brief, the induced ADSCs on the glass were washed with PBS for three times and fixed with 4% paraformaldehyde for 15 min. Next, the glass was stained with 0.2% Alizarin Red for 20 min. After washing with PBS, staining results were observed and taken photographs under optical microscope. The positive cells presented as nacarat.

For adipogenic differentiation of ADSCs, cells in passage 3 were incubated with adipogenic medium containing 10% FBS, 1 μM dexamethasone, 60 μM indomethacin, 500 nM IBMX, and 10 μg/m insulin in high-glucose DMEM medium. Oil Red O staining was used for quantitatively adipogenic assessments as per previous study. Briefly, cells were washed with PBS for twice and fixed using 4% paraformaldehyde for 5 min. Then, cells were washed again twice and stained in propylene glycol for 5 min, Oil Red O solution for 15 min at 60 °C. After Oil Red O solution was removed, cells were differentiated using 85% propylene glycol for 5 min. Next, cells were stained with hematoxylin for 30s. After washing again, results were observed and taken with optical microscopes.

### Isolation of exosome

Passage 3 ADSCs were seeded into 6-cm dish and incubated in a 37 °C incubator containing 5% CO2. When grown to 70–80% confluence, cells were cultured with fresh medium containing with exosome-free FBS for another 24 h which was obtained by ultracentrifugation at 100,000*g* for 18 h followed by filtration through a 0.22-μm filter. After 24 h of incubation, the conditioned medium (CM) was collected and exosome was isolated by utilizing exosome extraction kit (Wako pure Chemicals Industry, 293-77601) according to the manufacturer’s protocol. Briefly, 30 ml CM were centrifuged at 300×*g* for 5 min, 1200×*g* for 20 min, and then 10,000×*g* for 30 min at 4 °C to remove cells, debris, and large extracellular vesicles (EVs). Next, the CM were concentrated to 1 mL by using Amicon Ultra-15 Ultracel-100 K (Merck KGaA, Darmstadt, Germany, UFC910024). Then concentrated CM were incubated with Streptavidin Magnetic Beads (60 mg) accompanied by additional 1 μg of biotinylated mouse Tim4-Fc, 350 μL Exosome Capture Immobilizing Buffer, and 50 μL Exosome Binding Enhancer overnight at 4 °C. The next day, the beads were washed with washing buffer for three times and the bound EVs were eluted by utilizing Exosome Elution Buffer.

### Transmission electron microscopy (TEM)

Exosome morphology was analyzed using TEM analysis kit (E1610, Weihui Biotechnology, Peking, China). Firstly, exosome was placed on parafilm membrane as a 10 μL drop of exosome suspension and the EM membrane covered with formvar carbon was put up on the suspension for 10 min to adsorb exosome as much as possible. After washing with wash buffer, EM containing exosome was observed by transmission electron microscopy (JEOL, JSM-IT300LV) and images were taken using an electron sensitive Olympus KeenView CCD camera.

### Nanoparticle tracking analysis (NTA)

Exosome was diluted to a volume of 1 mL in TPM to analysis. Size and concentration of exosome were determined through Nanosight Tracking Analysis by utilizing ZetaView PMX 110 (Particle Metrix, Meerbusch, Germany) according to previous protocol [19].

### Urine protein, serum creatinine (Scr), and blood urea nitrogen (BUN) measurement

Urine was collected after 12 weeks of exosome treatment and measured using BCA kit. In brief, mixture of BCA working solution and urine was co-incubated at 37 °C for 30 min. The OD value was tested at the wavelength of 562 nm. In the end of exosome treatment, blood plasma was collected by abdominal aortic method and stand for 2 h at room temperature. After centrifugation at 3500 rpm for 5 min, Scr and BUN levels were measured through detection kits (Scr: C011-1, Jiangcheng Bio, Nanjing; BUN: C013-2, Jiangcheng, Nanjing), respectively.

### Plasmid construction and cell transfection

For luciferase reporter plasmids, Smad1-3′UTR-wt (wild type) and Smad1-3′UTR-mut (mutant) fragments were inserted into pYr-MirTarget vector. For Smad1-overexpressed plasmid construction, mus Smad1 open reading frame fragment was inserted into pcDNA3.1 vector. Next, recombinant plasmids were transferred into DH5α coli cells and screened by clone assay. PCR amplification primers were as follows: Smad1-3′UTR-wt, forward: GGTTCTTTTCCAACGCTATT, reverse: CACTTCAGAAAGACTATCAG; Smad1-3′UTR-mut, forward: TTTGTTTGTTTTTAATGAAGACGTTAATCGTTATGACATGCATAG, reverse: ATAACGATTAACGTCTTCATTAAAAACAAACAAAAAACCCATTCA. After sequencing, the reporter plasmids were extracted by utilizing plasmid extraction kit.

### Dual luciferase reporter assay

HEK-293 T cells were seeded on 24-well plate and incubated for 24 h. Then, cells were transfected with Smad1-3′UTR reporter and miR-486 mimic NC/miR-486 mimic plasmids for another 48 h. Culture medium was removed, and cells were washed with PBS for twice. Cells were lysed through adding PLA buffer, and luciferase activity was measured by utilizing dual luciferase reporter assay system (E1910, Promega). OD value was observed through microplate reader.

### Immunofluorescence (IF)

Cells were fixed in 4% paraformaldehyde for 30 min followed by washing for three times using PBS. Cells were treated with 0.1% Triton X-100 for 15 min and blocked using 5% BSA (Hyclone, SH30574.03) for 1 h. For renal tissue, specimen was fixed in 4% paraformaldehyde for 24 h or more. Through the production of paraffin sections and antigen retrieval, tissue sections were blocked using 5% BSA for 1 h. Then, cells and sections were incubated using primary against CD29 (sc-9970, 1:200, Santa Cruz), CD34 (bs-0646R, 1:500, bioss), CD44 (bs-4916R, 1:400, bioss), CD45 (bs-0522R, 1:300, bioss), CD90 (bs-0778R, 1:500, bioss), nephrin (sc-377246, 1:100, Santa Cruz), LC3 (12135-1-AP, 1:200, Proteintech) at 4 °C overnight. After incubation with secondary antibodies at 37 °C for 1 h, samples or cells were stained with DAPI for 5 min and laser confocal microscopy was adopted for observation and photo taking.

### Flow cytometry

ADSCs in passage 3 were digested by 0.25% trypsin, and cell suspension were filtered using 100-mesh filter. After 5 min of centrifugation, supernatant was removed and cells were resuspended with PBS followed by incubating with primary antibodies. Positive marker of ADSCs were including CD29 (1:500), CD44 (1:300), and CD90 (1:500). CD34 (1:300) and CD45 (1:300) were the negative marker of ADSCs. For exosome, the exosome suspension was used directly for purity identification by FACS according to previous study [20]. Positive marker of exosome, CD9 (bs-2489R, 1:100, Bioss), CD63 (GTX41877, 1:100, GeneTex), and CD81 (GTX41794, 1:100, GeneTex) were used for purity identification. In brief, exosomes were resuspended in 200 μL of PBS and aldehyde/sulfate beads (10 μL, Life Technologies) were added into exosome solution. After mixing for 15 min at room temperature by using a benchtop rotator, 600 μL PBS were added and incubated overnight at 4 °C. The next day, the pellets were resuspended in 40 μL of 2% BSA in PBS and stained with CD9, CD63, CD81, and their isotype control antibodies at RT for 30 min. After centrifugation at 12,000 rpm for 1 min, the pellets were resuspended in 20 μL BSA and stained with secondary antibodies. Eventually, the beads containing exosome were analyzed using the LSR Fortessa X-20 cell analyzer (BD Biosciences).

For apoptosis detection, cells were digested using 0.25% trypsin without EDTA and centrifuged at 1500 rpm for 5 min. The precipitate was resuspended and washed gently with PBS. Through 5 min of centrifugation at 1500 rpm, cells were suspended using 300 μL binding buffer. Cells were stained with 5 μL Annexin V-FITC in darkness for 5 min following with 10 μL PI staining for 10 min at room temperature. Then, cells apoptosis was measured by Flow cytometry (BD Biosciences, FACSCalibur).

### CCK8 assay and Tunel staining

MPC5 cells treated with high-glucose or ADSCs-Exo were seeded on 96-well plate at a density of 3000 cells/well. After 24 h, 48 h, 72 h, and 96 h of incubation, 100 μL CCK8 was added into cells following by 2 h of incubation. OD value was detected through microplate reader (Thermo, Multiskan MK3) at the wavelength of 450 nm.

For tissue apoptosis measurement, tissue sections were assessed according to the manufacturer’s instruction (Biobox, BA27). In brief, the specimens were stand in TUNEL reaction solution containing TdT and Streptavidin-FITC to label DNA fragments. After washing, the labeled preparations were incubated using POD-conjugated Anti-FITC at 37 °C for 30 min in darkness. Then, slides were added with DAB reaction solution and stained with DAPI followed by a step of hydrochloric acid alcohol differentiation, ethanol dehydration, xylol incubation, and mounting with neutral resins. The resulting images were processed and analyzed using microscope.

### RNA isolation and quantitative real-time polymerase chain reaction

Tissue and cell total RNA were extracted using TRIZOL reagent. One microgram RNA was used to perform reverse transcription by using First Strand cDNA Synthesis Kit (Fermentas, K1622) and polymerase chain reaction using SYBR Green qPCR kit (Thermo, K0222). Primers of miR-486 and internal control were as follows: miR486, TATAGCTCCTGTACTGAGCTGC; miR universal primer, CCAGTCTCAGGGTCCGAGGTATTC. U6 F, CTCGCTTCGGCAGCACA; U6 R, AACGCTTCACGAATTTGCGT.

### Western blotting

Protein of renal tissue and MPC5 cells were processed SDS-PAGE according to the previous study [21]. All primary antibodies were included CD9 (1:1000, Bioss, bs-2489R), CD63(1:1000, GeneTex, GTX41877), CD81 (1:800, GeneTex, GTX41794), β-actin (1:1000, CST, 4970), Caspase3 (1:1000, CST, 9662), mTOR (1:1000, CST, 2983), p-mTOR (1:500, CST, 5536), LC3 (1:500, Proteintech, 12135-1-AP), p62 (1:500, Proteintech, 18420-1-AP), Beclin1 (1:500, CST, 3495) and Smad1 (1:1000, Affinity, AF6451).

### Statistical analysis

Statistical analysis was processed by utilizing SPSS 21.0 software. Data were presented according to three independent experiments and appeared as mean ± standard error of the mean. Histogram and line chart were analyzed by GraphPad Prism 5.0 (GraphPad Software Inc., USA) using Student’s *t* test or One-way ANOVA analysis. *p* < 0.05 was regarded as significant difference.

## Results

### Adipose-derived stem cells (ADSCs) possesses strong differentiation capability

In order to obtain ADSCs-derived exosome, ADSCs were isolated from subcutaneous adipose tissue in groin of C57BL/KsJ db/m mice. At passage 3 of ADSCs, cell purity was assessed by performing IF staining using positive marker (including CD29, CD44 and CD90) and negative marker (including CD34 and CD45) of ADSCs. As shown in Additional file 1: Figure S1A, most of ADSCs expressed CD29 (red), CD44 (green), and CD90 (red) but not CD34 and CD45 (Additional file 1: Figure S1A). A relatively greater number of CD29-, CD44-, and CD90-positive cells were observed by flow cytometry in ADSCs. However, there are no CD34- and CD45-positive cells in ADSCs (Additional file 1: Figure S1B). Through performing flow cytometry and CCK8 assays, we determined that passage 6 of ADSCs had strong growth capacity (Additional file 1: Figure S2A–C). To confirm the differentiation ability of ADSCs, cells were cultured with osteogenic- and adipogenic medium. Next, ALP activity detection and Alizarin Red Staining results were the indicators of osteogenic capability of ADSCs which showed that osteogenic medium significantly induced the level of ALP and the number of Alizarin Red staining positive cells in ADSCs (Additional file 1: Figure S2D–E). Oil Red O staining results showed higher adipogenic potential under the condition of adipogenic medium (Additional file 1: Figure S2F). Thus, these data indicate that the ADSCs are isolated successfully.

### Adipose-derived stem cells are efficient producers of exosome

**Fig. 1:**
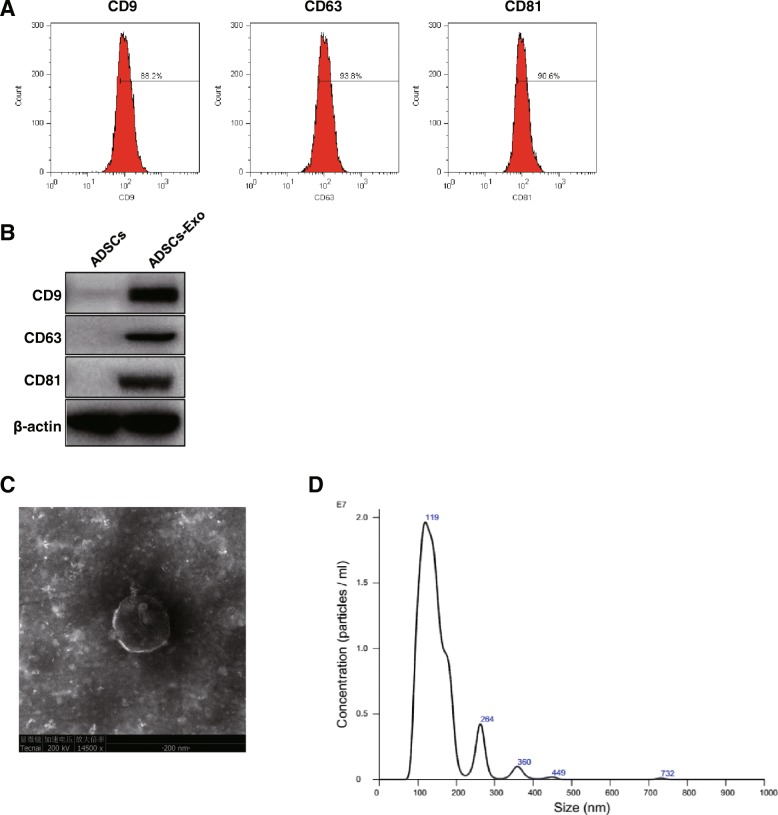
Authentication of ADSCs-derived exosome. **a** Purity identification of ADSCs-Exo by flow cytometry using CD9, CD63, CD81, and their isotype control antibodies. **b** Measurement of protein level of CD9, CD63, and CD81 by western blotting. **c** Images of exosome morphology taking by transmission electron microscopy (TEM). **d** Observation of number and size of exosome by nanoparticle tracking analysis. ADSCs-Exo indicates ADSCs-derived exosome

Extracellular vesicles (EV), term used as the exosome characteristics, were isolated from ADSCs to explore the role of ADSCs-Exo on podocyte damage. Firstly, we identified the purity of ADSCs-Exo by flow cytometry using immune-labelling method. The results showed that 89.3% of CD9-, 94.6% of CD63-, and 93.7% of CD81-positive particles in the isolated EV (Fig. 1a). Exosome surface markers CD9, CD63, and CD81 were also presented high expression in comparison with ADSCs (Fig. 1b). Through the observation using transmission electron microscopy, ADSCs-Exo appeared as circular particles (Fig. 1c). With NTA analysis, the size distribution of total extractive focused on the average size of 93.24 nm which was in accord with the feature of exosome (40–120 nm) (Fig. 1d). The total particles of ADSCs-exosome was found to be high, averaging around 1.73 × 10^8^/mL (Fig. 1d). Collectively, we successfully obtain exosome particles from ADSCs.

### ADSCs-Exo significantly attenuates pathologic symptom and cell apoptosis of spontaneous diabetes mice

**Fig. 2:**
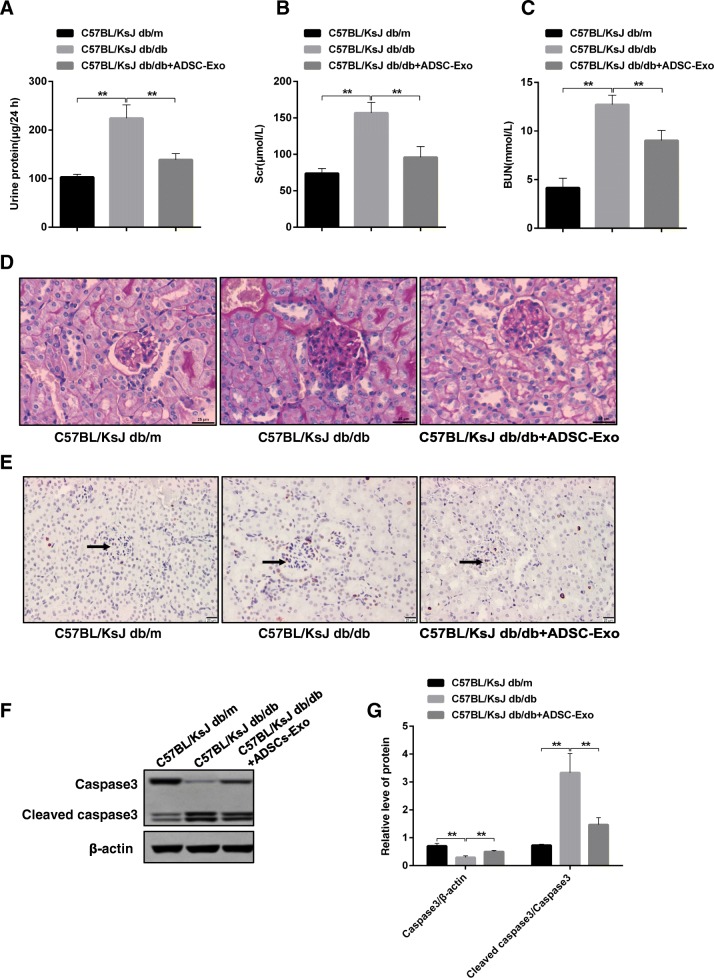
Effects of ADSCs-Exo on spontaneous diabetes mice. **a-c** Measurement of urine protein, Scr, and BUN levels in control and spontaneous diabetes mice before and after ADSCs-Exo treatment. **d** PAS staining of renal tissue section in three group mice. **e** Detection of apoptotic cells by Tunel staining in renal specimen. Black arrow indicates apoptotic cells. Brown color indicates positive cells. **f** Expression examination of caspase3 and cleaved-caspase3 in renal tissue by western blotting. **g** Relative quantitative analysis of caspase3 and cleaved-caspase3. Scale bar, 25 μm. **p < 0.01

Since USCs-derived exosome possesses protection effect on renal injury and podocyte apoptosis in rat, we try to explore whether ADSCs-Exo also have the same role as well as USCs-Exo. Firstly, in spontaneous diabetes mice, we found that levels of urine protein within 24 h (two-fold), Scr (three-fold), and BUN (three-fold) were robustly enhanced compared to C57BL/KsJ db/m (control group) (Additional file 1: Figure S3A–C). Besides, PAS staining results suggested that renal tissue had presented glomerular extracellular matrix (ECM) accumulation, glomerular mesangial cell proliferation, and base-membrane thickness in spontaneous diabetes mice compared to control mice indicating DN mice model was successfully established (Additional file 1: Figure S3D). Once administration with ADSC-Exo according to the flow chart in Additional file 1: Figure S3E, the increase of urine protein, Scr, and BUN were inhibited observably (Fig. 2a–c). Additionally, ADSCs-Exo administration significantly attenuated pathological changes in DN mice model (Fig. 2d, the right image vs the middle image). It is confirmed that podocyte apoptosis is the hallmark of DN. As you saw from our tunel staining result, the increase of cell apoptosis in C57BL/KsJ db/db was remarkably reduced under the treatment with ADSCs-Exo (Fig. 2e, the right image vs the middle image). Apoptotic signal protein such as cleaved caspase3 was promoted in DN mice, whereas suppressed in the present of ADSCs-Exo (Fig. 2f–g). These results demonstrate that ADSCs-Exo can relieve the pathology symptom in DN mice.

### ADSCs-Exo effectively inhibits high glucose-induced cell apoptosis in MPC5 cells

**Fig. 3:**
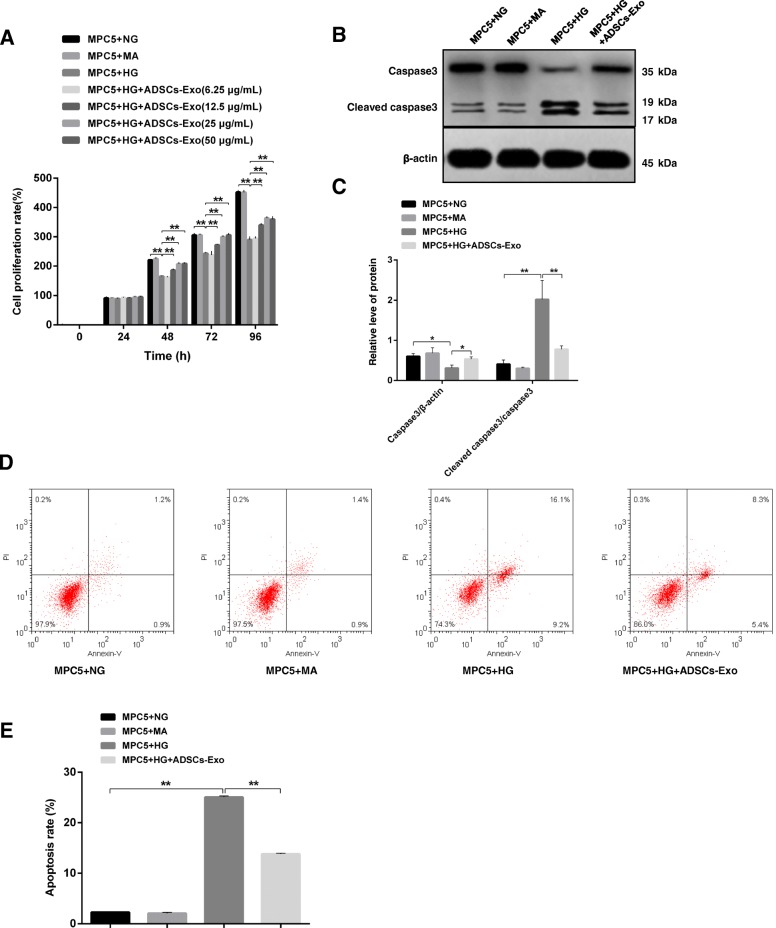
Impacts of ADSCs-Exo on high glucose-induced MPC5 cells apoptosis. **a** MPC5 cells viability was evaluated through CCK8 assay under the condition of normal glucose (NG), mannitol (MA), high glucose (HG), and concentration gradient ADSCs-Exo for 24 h, 48 h, 72 h, and 96 h. **b** Expression examination of caspase3 and cleaved-caspase3 in MPC5 cells by western blotting. **c** Relative quantitative analysis of caspase3 and cleaved-caspase3 in NG/MA/HG/ADSCs-Exo treated MPC5 cells. **d** Apoptotic MPC5 cells were determined by flow cytometry. **e** Relative apoptosis rate of MPC5 cells under the treatment of NG/MA/HG/ADSCs-Exo. NG indicates 5.5 mM d-glucose. MA indicates 5.5 mM d-glucose+ 24.5 mM mannitol. HG indicates 30 mM d-glucose. **p < 0.01

**Fig. 4:**
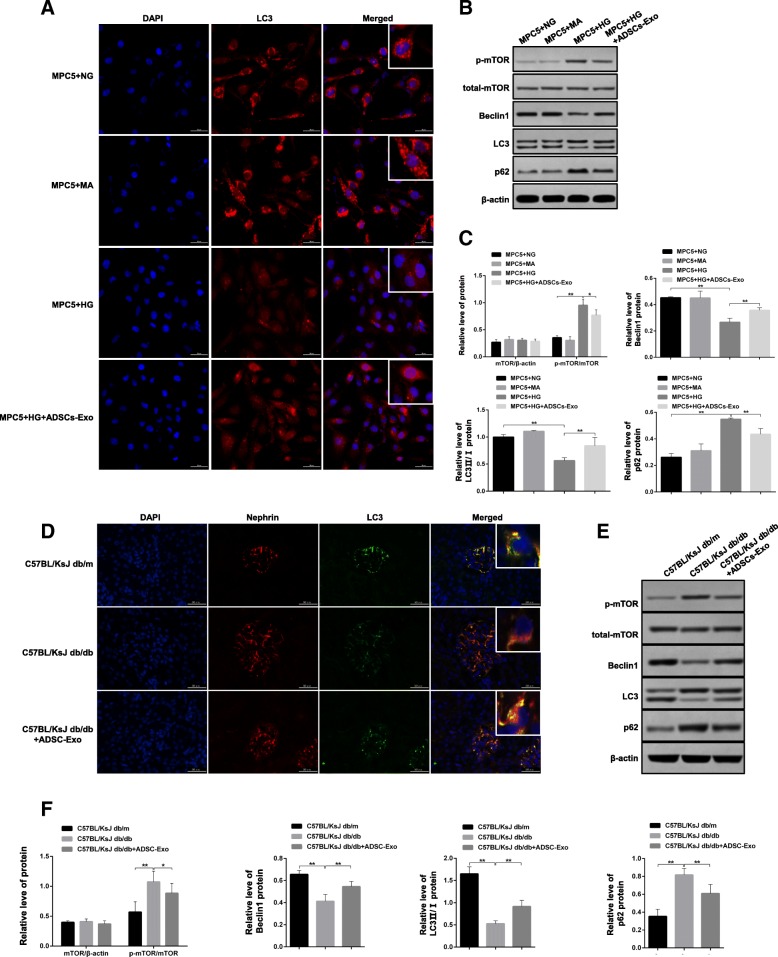
Evaluation of the role of ADSCs-Exo on high glucose-mediated the alteration of autophagy and mTOR signaling. **a** Measurement of LC3 protein level by IF staining in MPC5 cells. **b** Autophagy-related signaling molecules were evaluated by western blotting using antibodies against p-mTOR, mTOR, p62, Beclin1, and LC3 in MPC5 cells. **c** Relative quantitative analysis of protein levels in panel B. **d** Measurement of LC3 and nephrin protein level by IF staining in control and spontaneous diabetes mice before and after ADSCs-Exo treatment. **e** Autophagy-related signaling molecules were evaluated by western blotting using antibodies against p-mTOR, mTOR, p62, Beclin1, and LC3 in mice. **f** Relative quantitative analysis of protein levels in panel **e**. Scale bar, 50 μm. **p < 0.01

The protection effect of ADSCs-Exo on podocyte damage was not only found in DN mice. In mouse podocyte MPC5 cells, high glucose induced the cell growth inhibition compared in the control group (NG- and MA-treated cells). ADSCs-Exo treatment effectively relieved the negative effect of high glucose on MPC5 cells in concentration- and time-dependent manners (≤ 25 μg/mL), especially in the concentration of 25 μg/mL at 48 h (Fig. 3a). Through immunoblotting experiment, we found that 25 μg/mL of ADSCs-Exo vividly reduced the activity of cleaved caspase3 which showed high level under the condition of high glucose (Fig. 3b, c). In the analysis results of flow cytometry, high glucose-mediated the increase of apoptotic cell proportion was sharply reduced with the treatment of ADSCs-Exo (Fig. 3d, 16.1 + 9.2% vs 8.3 + 5.4%). Combined with the statistical result of apoptosis rate in Fig. 4e (Fig. 3e), we conclude that ADSCs-Exo also ameliorate high glucose-induced podocyte injury in vitro.

### Autophagy induction and mTOR signaling activation in high glucose-induced MPC5 cells and DN mice are suppressed by ADSCs-Exo treatment

Autophagy dysfunction always is the indicator of podocyte apoptosis/damage in vitro and in vivo [22]. Due to the improvement role of ADSCs-Exo on cell apoptosis in MPC5 cells and DN mice, whether ADSCs-Exo-mediated the protection effect was involved in the process of autophagy downregulation-induced cell apoptosis remained unclear and needed further investigation. LC3 was the common indicator protein of autophagosome. Once autophagy was downregulated, the rate of LC3 II/LC3 I was repressed which was contributed to the process of podocyte apoptosis. In the IF staining assay, high glucose obviously decreased the description of point aggregate of LC3 protein indicating autophagy reduction was involved in the process of HG-mediated cell apoptosis in MPC5 cells. But ADSCs-Exo administration significantly recovered the decrease of LC3 level (Fig. 4a). Numerous studies have confirmed that mTOR signaling and p62 level were the key regulator of autophagy in DN. After performing western blotting assay, we discovered that both phosphorylation of mTOR and p62 level were enhanced under the condition of high glucose followed by the decrease of the marker protein of autophagy such as Beclin1 and LC3II/I. Conversely, treatment with ADSCs-Exo inhibited mTOR and p62/LC3 signaling pathway and enhanced the expression of autophagy-related proteins (Fig. 4b, c). In DN mice, expression of LC3 in podocyte which was positively stained with nephrin was reduced obviously (Fig. 4d). Besides, increase of mTOR and p62/LC3 signaling transduction and decrease of autophagy marker expression were also observed in DN mice (Fig. 4e). However, ADSCs-Exo injection remarkably restored these changes which suggested that the restoration of autophagy was required in ADSCs-Exo-mediated the improvement of podocyte damage in MPC5 cells and DN mice (Fig. 4d, f).

### miR-486 is required in the process of ADSCs-Exo-mediated the injury protection in MPC5 cells

**Fig. 5:**
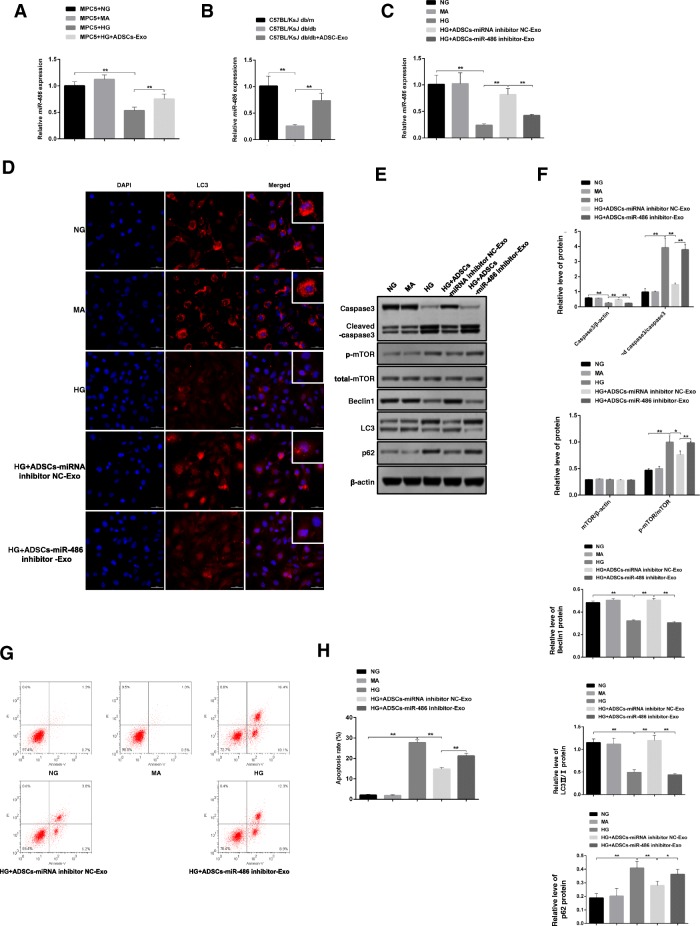
Exploration of the role of miR-486 on ADSCs-Exo-mediated damage inhibition in MPC5 cells. **a**, **b** Expression examination of miR-486 in ADSCs-Exo-treated MPC5 cells and renal tissue by qRT-PCR. **c** miR-486 expression was evaluated by qRT-PCR in HG-induced MPC5 cells treated with ADSCs-Exo NC and ADSCs-Exo-miR-486 inhibitor. **d** Measurement of LC3 protein level by IF staining in MPC5 cells treated with HG/MA/HG/ADSCs-Exo/ADSCs-Exo-miR-486 inhibitor. **e** Apoptosis- and autophagy-related molecules were determined by western blotting using primary antibodies against caspase3, cleaved-caspase3, p-mTOR, mTOR, p62, Beclin1, and LC3 in MPC5 cells. **f** Relative quantitative analysis of protein levels in panel **e**. **g** Apoptotic MPC5 cells were determined by flow cytometry under conditional treatment. **h** Relative apoptosis rate of MPC5 cells in panel **g**. Scale bar, 50 μm. *p < 0.05; **p < 0.01

In comparison to normal people, diabetic patients showed lower expression of miR-486 implying that miR-486 might be a critical regulator in the process of DN. To investigate the molecule mechanism of ADSCs-Exo-launched damage protection, we determined the expression pattern of miR-486 under the treatment with and without ADSCs-Exo. We found that the level of miR-486 was significantly downregulated in high glucose-induced MPC5 cells and DN mice, whereas the expression of miR-486 was recovered in the present of ADSCs-Exo in vitro and in vivo (Fig. 5a, b). These results motivated us to speculate whether miR-486 of ADSCs-Exo could affect high glucose-induced MPC5 cells and renal injury. Firstly, we verified the inhibition effect of miR-486 inhibitor. As shown in Additional file 1: Figure S4, miR-486 inhibitor transfection significantly repressed the level of miR-486 in ADSCs and ADSCs-Exo (Additional file 1: Figure S4A–C). Once the inhibition of the expression of miR-486 in ADSCs using miR-486 inhibitor, ADSCs-Exo-induced upregulation of miR-486 in the presence of HG was limited observably (Fig. 5c). To further explore the role of miR-486 on autophagy downregulation-mediated podocyte apoptosis in MPC5 cells, we evaluated the expression of autophagy- and apoptosis-related proteins in the present of miR-486 inhibitor. As you could see, ADSCs-Exo restored high glucose-induced decrease of LC3 level, but miR-486 inhibition in ADSCs dramatically blocked the protection role of ADSCs-Exo on LC3 expression in MPC5 cells (Fig. 5d). Under the same conditions, ADSCs-Exo mediated the upregulation of autophagy, including reducing high glucose-induced upregulation of mTOR and p62 signaling and the downregulation of Beclin1 and LC3II/I, was thoroughly suppressed through regulating the signaling molecules mentioned above (Fig. 5e–f). Besides autophagy, ADSCs-Exo-launched protective effect on cell damage was also neutralized in the presence of miR-486 inhibitor. For example, cleaved caspase3 showed the same level with HG-treated group in miR-486 inhibitor group indicating miR-486 inhibition in ADSCs which fully reversed the role of normal ADSCs-Exo (Fig. 5e, f). By performing flow cytometry, we also discovered that ADSCs-Exo mediated the decrease rate of apoptotic cells which weakened in the condition of miR-486 inhibition (Fig. 5g, h). These data indicate that miR-486 in ADSCs was needed in the course of ADSCs-Exo-mediated protective effect on cell injury through regulating autophagy-related signaling pathway.

### ADSCs-Exo ameliorates podocyte damage by regulating miR-486/Smad1/mTOR signaling pathway

**Fig. 6:**
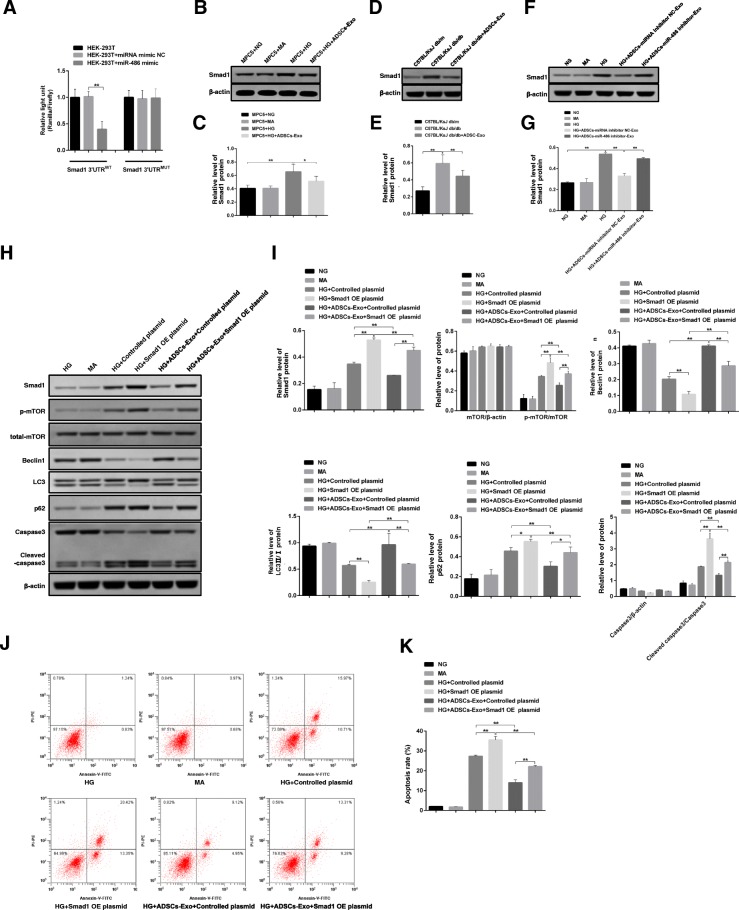
Mechanism exploration of ADSCs-Exo-mediated injury protection in DN and high glucose-induced apoptosis in MPC5 cells. **a** Luciferase activity of Smad1 was measured by utilizing dual luciferase reporter assay system. **b**, **c** Protein and relative quantitative analysis of Smad1 expression in MPC5 cells treated with NG, MA, HG, and ADSCs-Exo. **d**, **e** Protein and relative quantitative analysis of Smad1 expression in control and spontaneous diabetes mice before and after ADSCs-Exo treatment. **f**, **g** Protein and relative quantitative analysis of Smad1 expression in MPC5 cells treated with NG, MA, HG, ADSCs-Exo-NC, and ADSCs-Exo-miR-486 inhibitor. **h** Protein levels of Smad1, caspase3, cleaved-caspase3, p-mTOR, mTOR, p62, Beclin1, and LC3 in MPC5 cells treated with NG, MA, HG-NC, HG-Smad1, ADSCs-Exo, and ADSCs-Exo-Smad1. **i** Relative quantitative analysis of protein expression in panel **h**. **j** Apoptotic MPC5 cells were determined by flow cytometry. **k** Relative apoptosis rate of MPC5 cells in panel **j**. *p < 0.05; **p < 0.01

To explore the direct target of miR-486 in MPC5 cells, we screened the target gene of miR-486 using the online prediction software and found that miR-486 could directly bind to smad1’s 3′UTR region implying smad1 might be the specific target gene of miR-486 in MPC5 cells. Combined with dual luciferase reporter assay, we discovered that the luciferase reporter activity of smad1 3′UTR^WT^ was obviously inhibited in the group of miR-486 mimic-treated cells compared to miRNA mimic NC group. However, miR-486 mimic had no inhibition effect on cells transfected with smad1 3′UTR^MUT^ indicating smad1 was indeed the target gene of miR-486 (Fig. 6a). Besides, we also discovered that smad1 was upregulated in high glucose-induced MPC5 cells and DN mice. But ADSCs-Exo administration fully recovered the expression of smad1 in vitro and in vivo (Fig. 6b–e). Importantly, ADSCs-Exo derived from ADSCs treated with miR-486 inhibitor showed the same expression quantity of smad1 implying miR-486/smad1 signaling pathway was involved in ADSCs-Exo-launched injury repair (Fig. 6f, g). Based on these, we established the overexpressed construct of smad1 to further illustrate the role of miR-486/smad1 signaling on ADSCs-Exo-mediated damage repair. The overexpressed efficiency was verified through western blotting assay (Additional file 1: Figure S5A-B). It is confirmed that smad1 participates in the mTOR signaling pathway in many cases [23, 24]. In our study, we confirmed that HG induced the activation of mTOR/p62 signaling pathway and cleaved caspase3 expression; the inhibition of Beclin1 and LC3II/I were enlarged when smad1 was overexpressed in MPC5 cells (Fig. 6h). Though ADSCs-Exo could reverse these changes of signaling molecules by downregulating smad1, ADSCs-Exo had no effect on these molecules when smad1 were overexpressed in MPC5 cells (Fig. 6h, i). HG-induced apoptotic rate was attenuated by ADSCs-Exo treatment, but smad1 overexpression blunted the role of ADSCs-Exo (Fig. 6j, k). In a word, miR-486/smad1/mTOR might be the main signaling pathway in the process of ADSCs-Exo-mediated damage repair in MPC5 cells and DN mice.

## Discussion

Diabetic nephropathy as a serious microvascular complication of both type 1 and type 2 diabetes mellitus is expected to contribute to a significant percentage of the worldwide morbidity rate [25, 26]. Therefore, there is urgency to investigate novel therapeutic target to combat diabetic nephropathy. In the present study, we confirmed that ADSCs-Exo significantly prevented from renal injury by regulating autophagy homeostasis in podocyte. Maybe, ADSCs-Exo would be a new therapeutic target for DN treatment.

Transient presence of MSCs can accelerate the process of renal repair in mice through providing a paracrine support including the secretion of exosomes, growth factor, and cytokines [27, 28]. The evidence is mounting that exosomes derived from ADSCs act the core effects on protecting podocyte from conditioned medium-mediated damage [29, 30]. ADSCs-Exo overexpressing nuclear factor-E2-related factor 2 (Nrf2) promote wound healing and can be suitable for clinical application in the treatment of diabetic foot ulcers (DFU) [31]. In our research, we verified that ADSCs-Exo administration prevented renal injury from diabetes and the protective role of ADSCs was mainly based on its paracrine.

Podocytopathy such as podocyte damage has been regarded as the research focus in deciphering cellular and molecular mechanisms of DN [32]. Dysregulated cellular homeostasis is the hallmark of podocyte injury in DN. As catabolic process, autophagy dysfunction-mediated imbalance of homeostasis has been suggested to play pathogenic roles in podocyte/renal injuries in DN mice model with involving in many signal transduction pathways [33, 34]. Hyperactivation of the mTOR pathway in DN plays a pivotal role in the process of podocyte injury and the decline of glomerular filtration rates [35]. Damaged podocyte is always accompanied by the decrease of autophagy flux, the accumulation of p62, and the interaction between p62 and LC3 [36]. Therefore, mTOR and p62/LC3 signaling transduction are involved in the pathogenesis of autophagy dysfunction-mediated podocyte injury. Administration with ADSCs-Exo, activation of mTOR, and enhancement of p62 expression and LC3 II/LC3 I rate in high glucose-exposed MPC5 cells and DN mice were significantly inhibited indicating ADSCs-Exo-mediated increase of autophagy flux in damaged podocyte.

Stem cells-derived exosomes are able to transport bioactivators between cells through a new mechanism of cell-to-cell communication, including growth factors, mRNA, and miRNA [27, 37]. Based on this feature, exosomes have got a lot of attention with respect to drug delivery. MSC exosomes enriched in miR-146b can decrease tumor size in glioblastoma multiforme (GBM) by intratumoral injection [38]. Exosomes secreted from MSCs can also transfer miR-143 to GBM cells leading to migration and self-renewal inhibition [39]. Recently, microRNAs (miRNAs) in the extracellular environment have emerged as critical regulators in renal fibrosis, acute kidney injury, DN, and progressive kidney disease [40–42]. Especially, miRNAs packaged in extracellular vesicles such as exosomes showed concentration changes associated with DN occurrence and received significant attention as potential noninvasive biomarkers for diagnosis and treatment of DN [43]. In our study, miR-486 enriched in ADSCs-Exo induced the upregulation of miR-486 in MPC5 cells and DN mice. miR-486 inhibition in ADSCs significantly suppressed the protection role of ADSCs-derived exosome in high glucose-induced podocyte damage and autophagy reduction. Eventually, we confirmed that miR-486 could directly regulate Smad1 expression followed by the increase of mTOR-mediated autophagy flux. Our data demonstrated that ADSCs-Exo mediated the transport of miR-486 to podocyte and the upregulation of miR-486 suppressed renal injury by regulating smad1/mTOR signaling pathway.

## Conclusions

In conclusion, miR-486 carried by ADSCs-Exo could transfer to podocyte functioning as an activator of autophagy and relieve cell damage in high glucose-induced MPC5 cell and DN mice. Potentially, exosomes secreted from ADSCs can be served as an excellent candidate for DN treatment.

## Additional file

Additional file 1: **Figure S1.** Isolation of ADSCs. (A-B)Purity identification of ADSCs by IF staining and flow cytometryusing CD29, CD34, CD44, CD45 and CD90 antibodies. **Figure S2.** Evaluation of cell proliferation and differentiation abilities of ADSCs. (A) Cell viability in passages 3, 6 and 9 of ADSCs were determined by flow cytometry. (B) Cell rate of G0/G1, S and G2/M phase in passages 3, 6 and 9 of ADSCs. (C) Cell viability in passages 3, 6 and 9 of ADSCs were determined by CCK8. (D) APL activity before and after osteogenic medium treatment. (E) Calcium deposit detection by PAS staining before and after osteogenic medium treatment. (F) Adipogenic assessments by oil red O staining after adipogenic induction medium treatment. Scale bar, 20 μm. **, *p* < 0.01. **Figure S3.** Authentication of the establishing of DN mice. (A-C) Measurement of urine protein, Scr and BUN in control and spontaneous diabetes mice. (D) PAS staining of renal tissue section in three group mice pretreatment with ADSCs-Exo. (E) Flow chart of exosome injection in control and spontaneous diabetes mice. At 13-weeks old, control and spontaneous diabetes mice were injected with PBS or ADSCs-Exo for additional 12 weeks. **, *p* < 0.01. **Figure S4.** Authentication of transfection efficiency of miR-486 inhibitor in ADSCs. (A) miR-486 expression was detected by qPCR after transfection for 24h in control ADSC, ADSC-miR486 inhibitor NC and ADSC-miR486 inhibitor. (B) ADSCs-Exo isolated from the above three group ADSCs were identified by WB using CD9, CD63 and CD81 antibodies. (C) miR-486 expression was detected by qPCR in ADSCs-Exo isolated from the above three group ADSCs. **, *p* < 0.01. **Figure S5.** Authentication of transfection efficiency of smad1 in MPC5 cells. Protein (A) and transcriptional level (B) of smad1 in MPC5 was identified through WB and qPCR methods after transfection for 24h. **p < 0.01. (PPTX 3080 kb)

## Abbreviations

ADSC-Exo: ADSCs-derived exosome
ADSCs: Adipose-derived stem cells
ALP: Alkaline phosphatase
AMPK: AMP-activated protein kinase
BUN: Blood urea nitrogen
DFU: Diabetic foot ulcers
DN: Diabetic nephropathy
EVs: Extracellular vesicles
GBM: Glomerular basement membrane
IBMX: 3 Isobutyl 1 methylxanthine
MSCs: Mesenchymal stem cells
mTOR: Mammalian target of rapamycin
Nrf2: Nuclear factor-E2-related factor 2
NTA: Nanosight tracking analysis
PAS: Periodic acid-Schiff
Scr: Serum creatinine
Smad1: Mothers against decapentaplegic homolog 1
TEM: Transmission electron microscopy
USCs: Urine-derived stem cells
