# Supplementary figures and images for: Exosome secreted from adipose-derived stem cells attenuates diabetic nephropathy by promoting autophagy flux and inhibiting apoptosis in podocyte
Source: Stem Cell Res Ther. 2019 Mar 15;10:95. doi: 10.1186/s13287-019-1177-1 (PMC6419838; doi:10.1186/s13287-019-1177-1)

## Slide 1
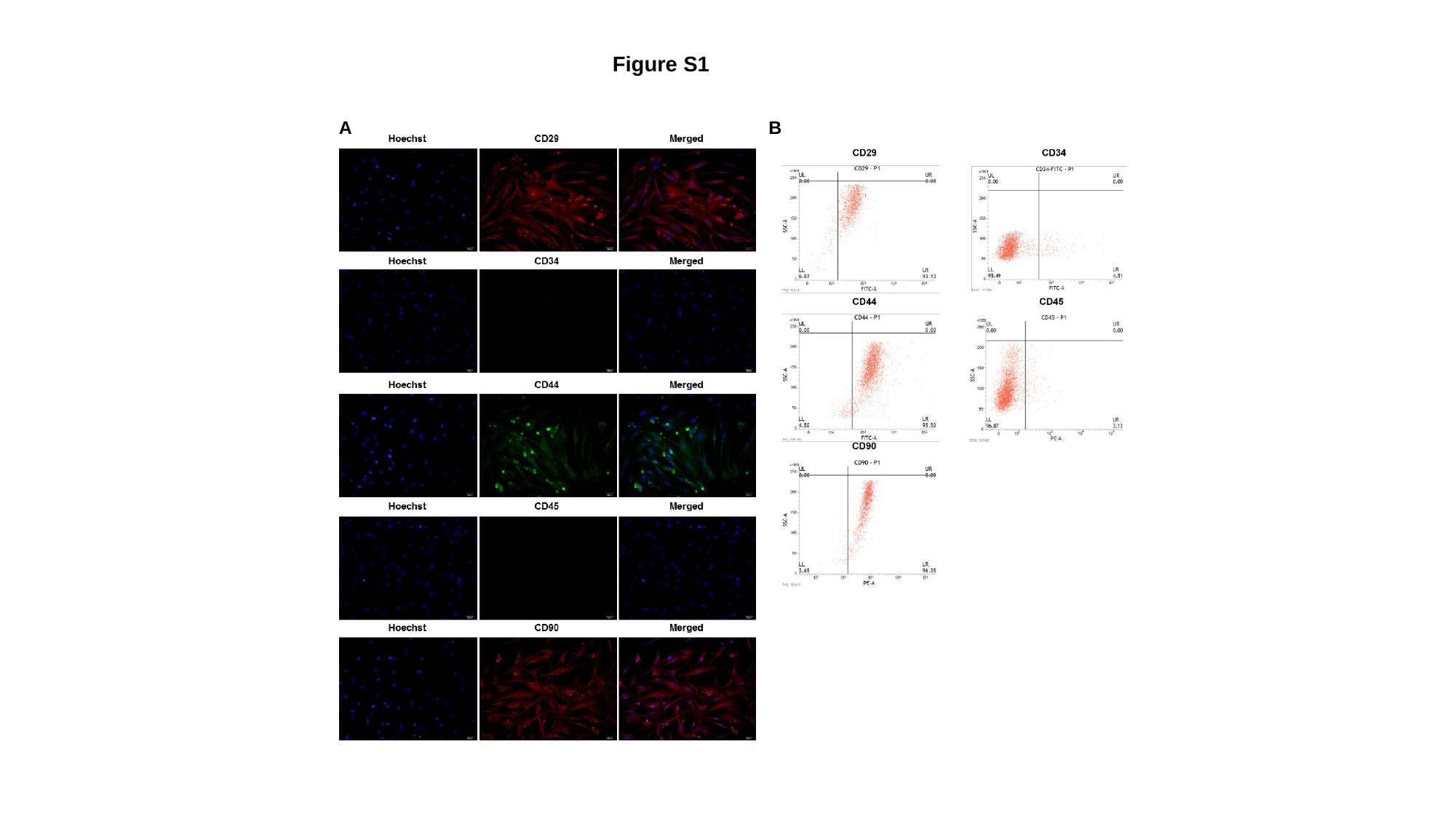

Figure S1
A
B

## Slide 2
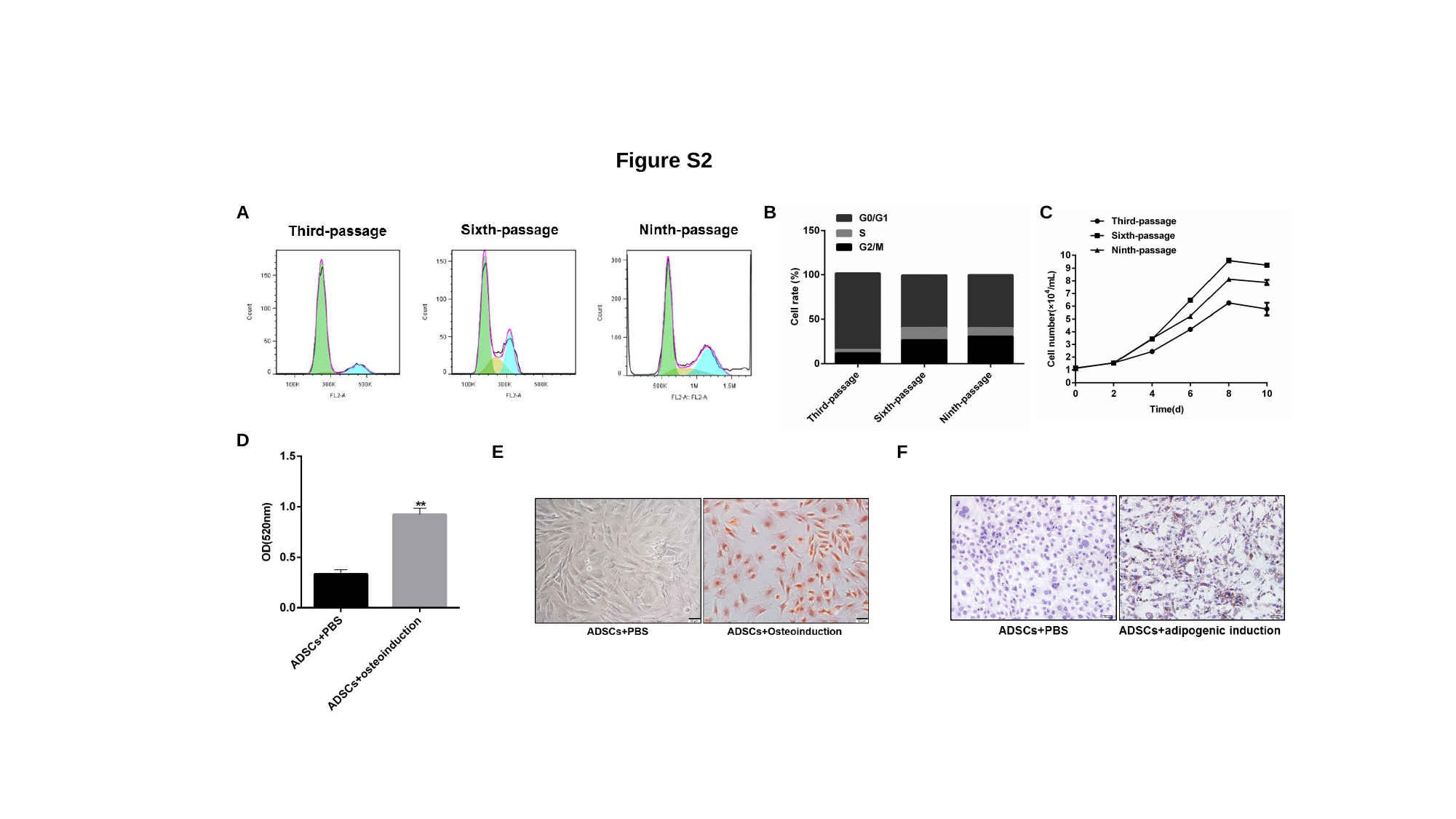

Figure S2
C
A
B
D
E
F

## Slide 3
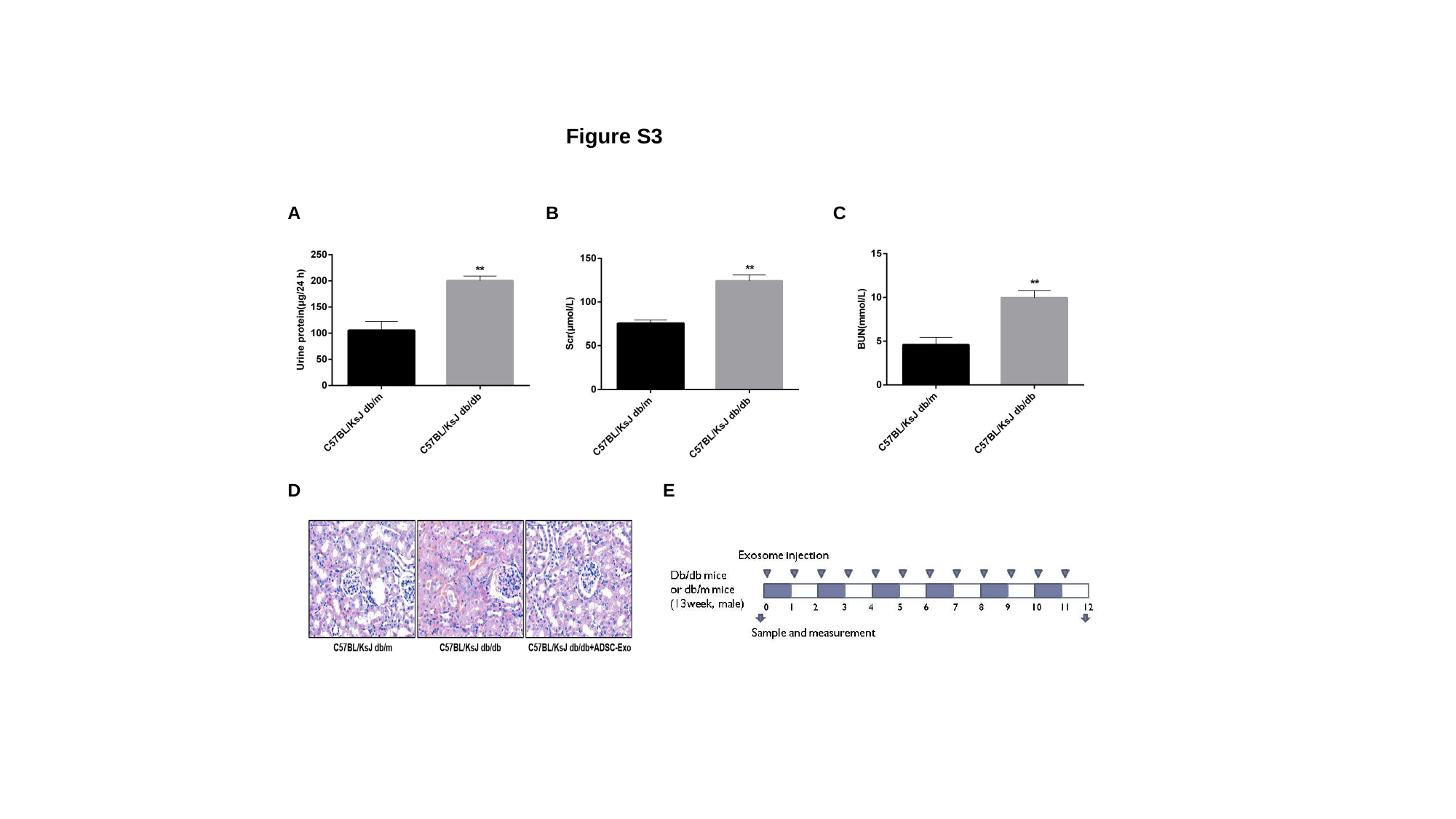

Figure S3
C
A
B
D
E

## Slide 4
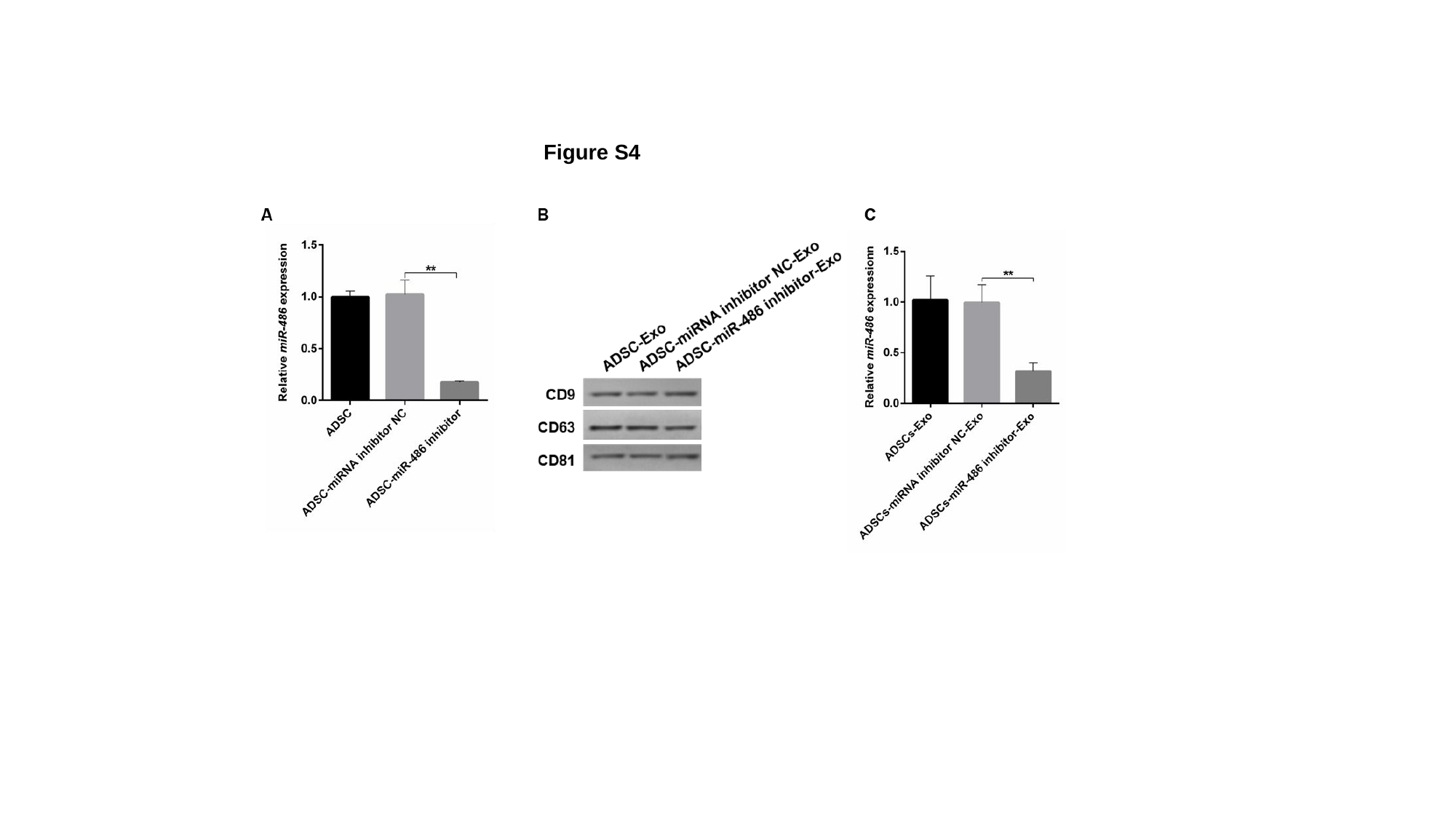

Figure S4

## Slide 5
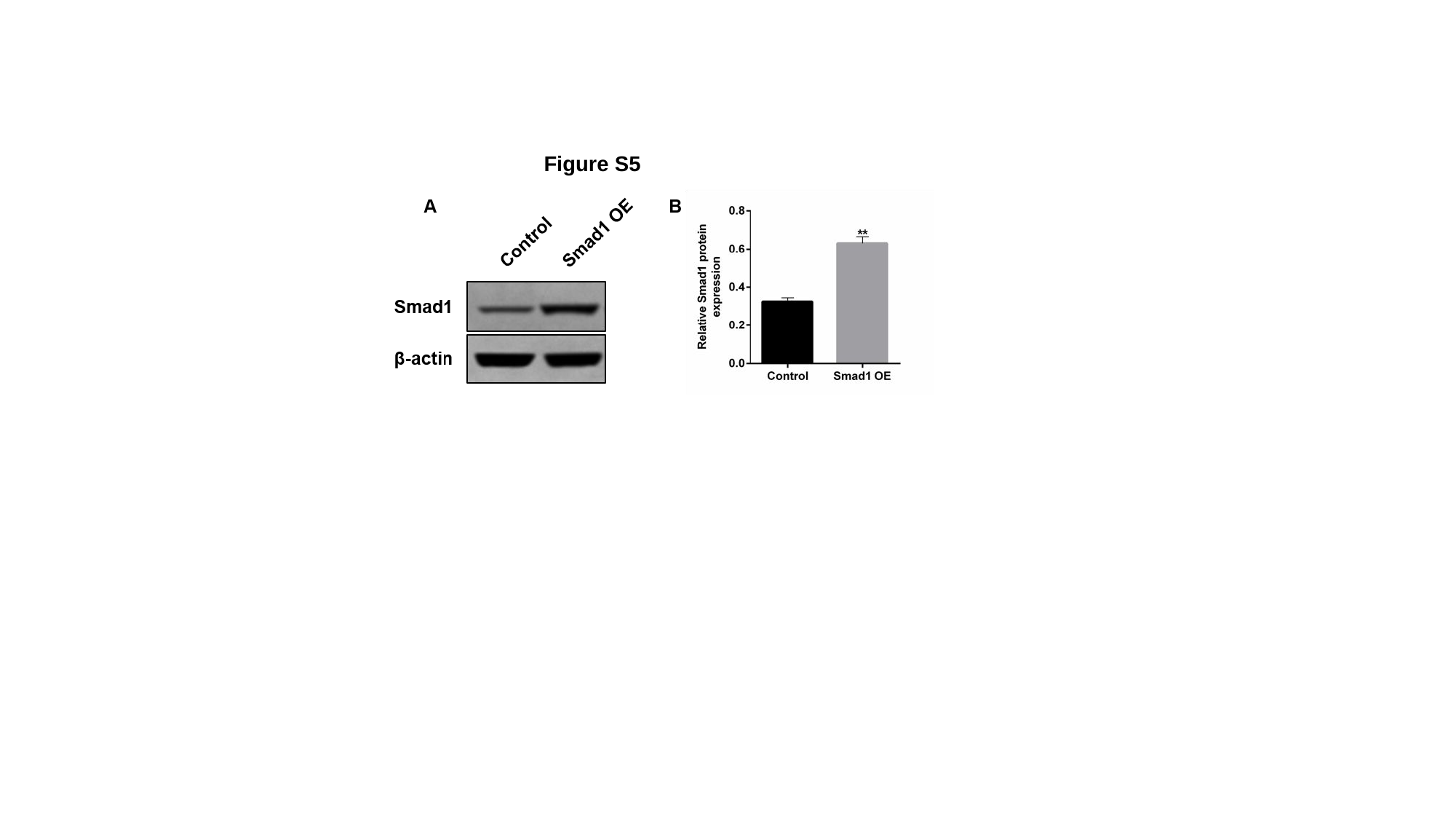

Figure S5

Supplement: Supplementary file 1 — Figure S1. Isolation of ADSCs. (A-B)Purity identification of ADSCs by IF staining and flow cytometryusing CD29, CD34, CD44, CD45 and CD90 antibodies. Figure S2. Evaluation of cell proliferation and differentiation abilities of ADSCs. (A) Cell viability in passages 3, 6 and 9 of ADSCs were determined by flow cytometry. (B) Cell rate of G0/G1, S and G2/M phase in passages 3, 6 and 9 of ADSCs. (C) Cell viability in passages 3, 6 and 9 of ADSCs were determined by CCK8. (D) APL activity before and after osteogenic medium treatment. (E) Calcium deposit detection by PAS staining before and after osteogenic medium treatment. (F) Adipogenic assessments by oil red O staining after adipogenic induction medium treatment. Scale bar, 20 μm. **, p < 0.01. Figure S3. Authentication of the establishing of DN mice. (A-C) Measurement of urine protein, Scr and BUN in control and spontaneous diabetes mice. (D) PAS staining of renal tissue section in three group mice pretreatment with ADSCs-Exo. (E) Flow chart of exosome injection in control and spontaneous diabetes mice. At 13-weeks old, control and spontaneous diabetes mice were injected with PBS or ADSCs-Exo for additional 12 weeks. **, p < 0.01. Figure S4. Authentication of transfection efficiency of miR-486 inhibitor in ADSCs. (A) miR-486 expression was detected by qPCR after transfection for 24h in control ADSC, ADSC-miR486 inhibitor NC and ADSC-miR486 inhibitor. (B) ADSCs-Exo isolated from the above three group ADSCs were identified by WB using CD9, CD63 and CD81 antibodies. (C) miR-486 expression was detected by qPCR in ADSCs-Exo isolated from the above three group ADSCs. **, p < 0.01. Figure S5. Authentication of transfection efficiency of smad1 in MPC5 cells. Protein (A) and transcriptional level (B) of smad1 in MPC5 was identified through WB and qPCR methods after transfection for 24h. **p < 0.01. (PPTX 3080 kb) [file 13287_2019_1177_MOESM1_ESM.pptx]
